# Comparison of the pharmacokinetics between L-BPA and L-FBPA using the same administration dose and protocol: a validation study for the theranostic approach using [^18^F]-L-FBPA positron emission tomography in boron neutron capture therapy

**DOI:** 10.1186/s12885-016-2913-x

**Published:** 2016-11-08

**Authors:** Tsubasa Watanabe, Yoshihide Hattori, Youichiro Ohta, Miki Ishimura, Yosuke Nakagawa, Yu Sanada, Hiroki Tanaka, Satoshi Fukutani, Shin-ichiro Masunaga, Masahiro Hiraoka, Koji Ono, Minoru Suzuki, Mitsunori Kirihata

**Affiliations:** 1 Kyoto University Research Reactor Institute, 2-1010 Asashiro-Nishi, Kumatori-cho, Sennan-gun, Osaka, 590-0494 Japan; 2 Department of Radiation Oncology and Image-Applied Therapy, Kyoto University Graduate School of Medicine, Yoshida-Konoe-cho, Sakyo-ku, Kyoto, 606-8501 Japan; 3 Research Center of Boron Neutron Capture Therapy, Research Organization for the 21st Century, Osaka Prefecture University, 1-1 Gakuen-cho, Nakaku, Sakai, Osaka Japan

**Keywords:** Boron neutron capture therapy, Boron concentration, L-BPA, FBPA, [^18^F]-L-FBPA

## Abstract

**Background:**

Boron neutron capture therapy (BNCT) is a cellular-level particle radiation therapy that combines the selective delivery of boron compounds to tumour tissue with neutron irradiation. L-*p*-Boronophenylalanine (L-BPA) is a boron compound now widely used in clinical situations. Determination of the boron distribution is required for successful BNCT prior to neutron irradiation. Thus, positron emission tomography with [^18^F]-L-FBPA, an ^18^F-labelled radiopharmaceutical analogue of L-BPA, was developed. However, several differences between L-BPA and [^18^F]-L-FBPA have been highlighted, including the different injection doses and administration protocols. The purpose of this study was to clarify the equivalence between L-BPA and [^19^F]-L-FBPA as alternatives to [^18^F]-L-FBPA.

**Methods:**

SCC-VII was subcutaneously inoculated into the legs of C3H/He mice. The same dose of L-BPA or [^19^F]-L-FBPA was subcutaneously injected. The time courses of the boron concentrations in blood, tumour tissue, and normal tissue were compared between the groups. Next, we administered the therapeutic dose of L-BPA or the same dose of [^19^F]-L-FBPA by continuous infusion and compared the effects of the administration protocol on boron accumulation in tissues.

**Results:**

There were no differences between L-BPA and [^19^F]-L-FBPA in the transition of boron concentrations in blood, tumour tissue, and normal tissue using the same administration protocol. However, the normal tissue to blood ratio of the boron concentrations in the continuous-infusion group was lower than that in the subcutaneous injection group.

**Conclusions:**

No difference was noted in the time course of the boron concentrations in tumour tissue and normal tissues between L-BPA and [^19^F]-L-FBPA. However, the administration protocol had effects on the normal tissue to blood ratio of the boron concentration. In estimating the BNCT dose in normal tissue by positron emission tomography (PET), we should consider the possible overestimation of the normal tissue to blood ratio of the boron concentrations derived from the values measured by PET on dose calculation.

**Electronic supplementary material:**

The online version of this article (doi:10.1186/s12885-016-2913-x) contains supplementary material, which is available to authorized users.

## Background

Boron neutron capture therapy (BNCT) is based on the nuclear reaction of the ^10^B nucleus, which absorbs low-energy neutrons to produce two high-linear-energy transfer particles (^4^He, 163 keV/μm and ^7^Li, 210 keV/μm) from the ^10^B(n, α)^7^Li reaction. The track ranges of these particles (5–9 μm) are limited to the diameter of a single cell. Theoretically, therefore, if ^10^B atoms are selectively accumulated in each tumour cell, and then the volume including the tumour is exposed to a sufficiently high fluence of thermal neutrons, targeting single tumour cells with less normal tissue damage than conventional therapy is possible [1]. Two boron compounds, disodium mercaptoundecahydrododecaborate (Na_2_B_12_H_11_SH; BSH) [2] and L-*p*-boronophenylalanine (L-BPA) [3], have been clinically utilized for the treatment of patients with various types of X-ray-refractory cancers such as malignant melanoma [4], malignant brain tumours [5–9], recurrent head and neck cancer [10, 11], and malignant mesothelioma [12]. In contrast to other types of radiotherapy, the cytocidal effect of BNCT on tumour tissue depends on the selective accumulation of boron compounds in the tumour. Thus, imaging methods that can illuminate the distribution of boron compounds noninvasively are important to forecast the efficacy of BNCT.

**Fig. 1:**
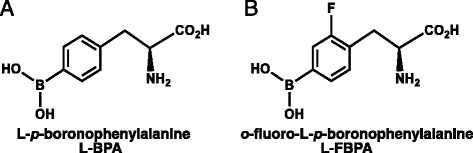
Chemical structure of L-BPA and its fluorine derivative for positron emission tomography. **a** L-BPA = L-*p*-boronophenylalanine, **b** L-FBPA = *o*-fluoro-L-*p*-boronophenylalanine

*o*-[^18^F]-Fluoro-L-*p*-boronophenylalanine ([^18^F]-L-FBPA) is an ^18^F-labelled radiopharmaceutical analogue of L-BPA used as a positron emission tomography (PET) probe to estimate the L-BPA distribution in BNCT using L-BPA prior to treatment [13]. This “theranostic approach”, which combines diagnostic modalities such as PET imaging and a therapeutic procedure such as neutron irradiation with boron compounds, is useful to confirm the irradiation dose from the boron neutron capture reaction and calculate the estimated tumour dose beforehand to assure safety and efficacy. However, the method used to evaluate the boron concentrations predicted by [^18^F]-L-FBPA PET for L-BPA accumulation in both tumour tissue and normal tissue has not been fully established because of several uncertain differences between [^18^F]-L-FBPA and L-BPA. First, the chemical structure and resulting physicochemical properties of [^18^F]-L-FBPA are not completely the same as L-BPA (Fig. 1). Actually, the degree of solubility in water and affinity to L-BPA antibodies were different between L-BPA for BNCT and [^18^F]-L-FBPA in a PET study. The lipophilic feature of fluorine can affect the distribution in tissues in the biological viewpoint. Lipid solubility of [^18^F]-L-FBPA is higher than that of L-BPA due to the fluorine atom of [^18^F]-L-FBPA. Second, the administered dose, solubilized form of compounds with water, and measurement methods for the boron concentrations are different between L-BPA and [^18^F]-L-FBPA. A high dose of L-BPA (250–900 mg/kg body weight) is administered as the L-BPA-fructose complex to increase the solubility in clinical situations [14], while a tracer dose of [^18^F]-L-FBPA is used in [^18^F]-L-FBPA PET without the formulation of the sugar complex. Continuous therapeutic dose of infusion has the possibility to make pharmacokinetic equilibrium saturate and alter the distribution of boron compounds in tissues compared to single shot tracer dose of injection. The amount of accumulated [^18^F]-L-FBPA is mainly measured by PET imaging because no other modality can detect tracer doses of the compound, while L-BPA concentrations are measured by other methods such as prompt gamma ray spectroscopy and inductively coupled plasma atomic emission spectroscopy (ICP-AES). Third, L-BPA is administered by continuous drip infusion over a period of more than 3 h; in contrast, [^18^F]-L-FBPA is administered as a single injection.

Some of these issues regarding the differences between L-BPA and [^18^F]-L-FBPA have already been examined. Regarding tumour tissue, Imahori showed that the boron level in the tumour tissue estimated with [^18^F]-L-FBPA PET method was closed to that of surgical specimens directly measured after a single injection of L-BPA with the prompt gamma method [15]. Regarding normal tissue, Hanaoka and colleagues demonstrated that each PET parameter of normal tissue after a tracer-dose single injection of [^18^F]-L-FBPA showed good correlation with the boron concentrations of normal tissue measured by ICP-AES after a single injection of L-BPA [16]. However, there are no previous reports that compare the difference of boron accumulation in tissues between [^18^F]-L-FBPA and L-BPA under the same injection dose, the same administration protocol, and the same boron measurement protocol. To show the validity of [^18^F]-L-FBPA PET as an accurate prediction tool to estimate the efficacy of BNCT with L-BPA before treatment, the remaining clinical questions should be addressed. First, what is the effect of the different injected doses of L-BPA and [^18^F]-L-FBPA? Do the different solubilized boron compounds (sugar-complex or not) affect the boron concentrations in tumour and normal tissues? Is there any other difference in the boron concentration in hollow viscus, in which the accumulation of the PET tracer is usually underestimated by PET imaging, and what is the difference in the normal tissue to blood ratio (N/B ratio), tumour to blood ratio (T/B ratio), or tumour to normal tissue ratio (T/N ratio) of boron concentrations between the continuous-infusion protocol used clinically and the single-injection protocol with PET analysis?

To answer these questions, we synthesized the cold stable isotope of [^18^F]-L-FBPA, [^19^F]-L-FBPA, using a procedure for the large-scale synthesis of L-FBPA that we previously developed because the chemical properties of [^19^F]-L-FBPA and [^18^F]-L-FBPA are identical and ^18^F is not necessary if the amount of L-FBPA in tissues is measured by ICP-AES. In this study, we examined the differences in the boron concentrations of the tumour tissue and normal tissues between L-BPA and [^19^F]-L-FBPA instead of [^18^F]-L-FBPA using the same dose, the same boron measuring methods (ICP-AES), and the same administration protocol as that for a subcutaneous tumour mouse model. Next, we evaluated the difference in the administration protocols between the single-injection mouse model and the continuous-infusion mouse model to clarify the remaining questions concerning the validation of the [^18^F]-L-FBPA PET study for BNCT treatment.

## Methods

### Materials

Optically pure [^19^F]-L-FBPA was synthesised by a hybrid process using L-aminoacylase from commercially available 2-fluoro-4-dihydroxyborylbenzaldehyde (Additional file 1: Figure S1). The water solubility of L-BPA and [^19^F]-L-FBPA were measured from supernatant of saturated solution by UV absorption method. SCC-VII, a squamous cell carcinoma cell line derived from C3H/He mice (Department of Radiology, Kyoto University), was maintained in Eagle’s minimum essential medium supplemented with 12.5 % foetal bovine serum and penicillin/streptomycin (100 U/mL). The cells were cultured at 37 °C with 5 % CO_2_.

Four-week-old female C3H/He mice were purchased from CLEA Japan (Tokyo, Japan). SCC-VII cells (3.0 × 10^5^) were subcutaneously inoculated into the left hind legs of C3H/He mice. Experiments were carried out 2 weeks after the inoculation, when each tumour had reached approximately 1 cm in diameter. All of the animal experiments were approved by the Animal Research Committee of Kyoto University Research Reactor Institute and were performed in accordance with the institutional laboratory animal handling guidelines and the guidelines governing animal care in Japan. The mice were handled according to the recommendations for the Handling of Laboratory Animals for Biomedical Research, compiled by the Committee on Ethical Handling Regulations for Laboratory Animal Experiments, Kyoto University.

### Preparation of L-BPA-fructose and [^19^F]-L-FBPA-fructose solution

L-BPA or [^19^F]-L-FBPA and fructose were dissolved in distilled water at a molar ratio of 1:1.5, and then a 1.15 M ratio of 1 N NaOH was added. Here, L-BPA or [^19^F]-L-FBPA was mixed with fructose to increase the solubility [14]. The mixture was stirred until L-BPA or [^19^F]-L-FBPA had completely dissolved, and the pH value was titrated to 7.6 with 1 N HCl. The solution was filtered through a 0.22-μm syringe filter for sterilization (Merck Millipore, Massachusetts, USA). The final concentration was set to 30 mg/ml.

### Experiment 1: comparison of the pharmacokinetics of L-BPA and [^19^F]-L-FBPA subcutaneously injected

L-BPA-fructose or [^19^F]-L-FBPA-fructose solution (500 mg/kg body weight) was subcutaneously injected into mice into the nuchal sites after a 3-h fast [17]. At each time point (30 min, 1, 2, 3, and 4 h), the boron concentrations in the blood, normal tissue, and tumour tissue were evaluated. Five mice were used for each time point. Totally 25 mice were used in this experiment. The 20 % trimmed mean, which was defined as the mean after discarding samples at the high and low end, was used to evaluate the difference between L-BPA and [^19^F]-L-FBPA in order to improve non-normality and skew in population distribution in this study. Time courses of the boron compounds in blood and tissues were defined as bioequivalent if the 90 % confidence intervals for the ratios of the average values of parameters fall within the acceptance limits of 0.80–1.25 for the maximum concentration and areas under the blood boron concentration curve (AUC) [18].

### Experiment 2: comparison of the pharmacokinetics of L-BPA and [^19^F]-L-FBPA continuously administered

L-BPA-fructose or [^19^F]-L-FBPA-fructose solution (500 mg/kg body weight) was continuously and subcutaneously injected at a constant rate of drip infusion into the nuchal sites after a 3-h fast using a syringe pump (KDS100; Muromachi Kikai Co., Ltd., Tokyo, Japan) without anaesthesia. Totally seven mice (four mice of L-BPA group and three mice of [^19^F]-L-FBPA group) were used in this experiment. First, each fructose solution of boron compound was continuously infused at a rate of 200 mg/kg body weight/h for 2 h. Next, the infusion rate was reduced to 100 mg/kg body weight/h for 1 h as in the clinical protocol for human patients previously reported [19]. After 3 h from the onset of drip infusion, tissue samples were excised immediately, and the boron concentrations in the blood, normal tissue, and tumour tissue were measured.

### Measurements of the boron concentration

Blood and tissue samples were digested with perchloric acid (60 %) and hydrogen peroxide (30 %) for 24 h at 75 °C. The boron concentration in each sample was determined by ICP-AES (iCAP 6000; Thermo Fisher Scientific Inc., Massachusetts, USA) and was normalized as μg/g. The N/B, T/B, and T/N ratios were calculated, and the 20 % trimmed mean values of each ratio were used for statistical analysis.

### Statistical analysis

The data are expressed as the mean ± standard deviation. The differences between the L-BPA group and [^19^F]-L-FBPA group and the subcutaneous injection group and continuous injection group were analysed by Welch’s *t*-test. The differences of N/B, T/B, and T/N ratio between the values 3 h after a single injection in Experiment 1 and the values after continuous drip infusion in Experiment 2 were analysed by a non-parametric ANOVA. *P* values less than 0.05 were considered to be statistically significant. All of the statistical analyses were performed using JMP Pro 11 software (SAS Institute Inc., Cary, NC, USA).

## Results

### Properties of L-BPA and [^19^F]-L-FBPA

The properties of L-BPA and [^19^F]-L-FBPA are shown in Table 1. The melting point and solubility in water of L-BPA were different from those of [^19^F]-L-FBPA. The acid dissociation constant of the amino group (pKb1) was also different between L-BPA and [^19^F]-L-FBPA.

**Table 1:** Physicochemical property of L-BPA and [^19^F]-L-FBPA

	L-BPA	[^19^F]-L-FBPA
Melting point	285–298 °C	266–269 °C
Solubility (water, 25 °C)	1.6 g/L	2.6 g/L
Acid dissociation constant	pKa: 2.10 (−COOH)	pKa: 2.11 (−COOH)
	pKb1: 8.43 (−NH_2_)	pKb1: 7.71 (−NH_2_)
	pKb2: 9.79 (−B(OH)_2_)	pKb2: 9.49 (−B(OH)_2_)

### Experiment 1: comparison of the pharmacokinetics of L-BPA and [^19^F]-L-FBPA subcutaneously injected

**Fig. 2:**
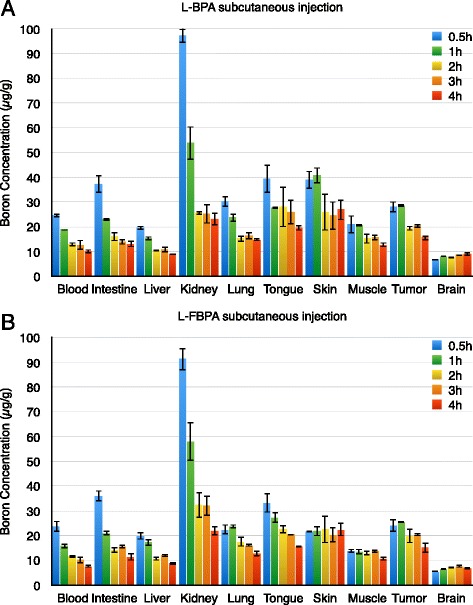
Transition of the boron concentrations in blood, normal tissue, and tumour tissue. This figure shows the transition of the boron concentrations in blood, normal tissue, and tumour tissue. **a** Boron concentrations after L-BPA subcutaneous injection. **b** Boron concentrations after [^19^F]-L-FBPA subcutaneous injection

**Fig. 3:**
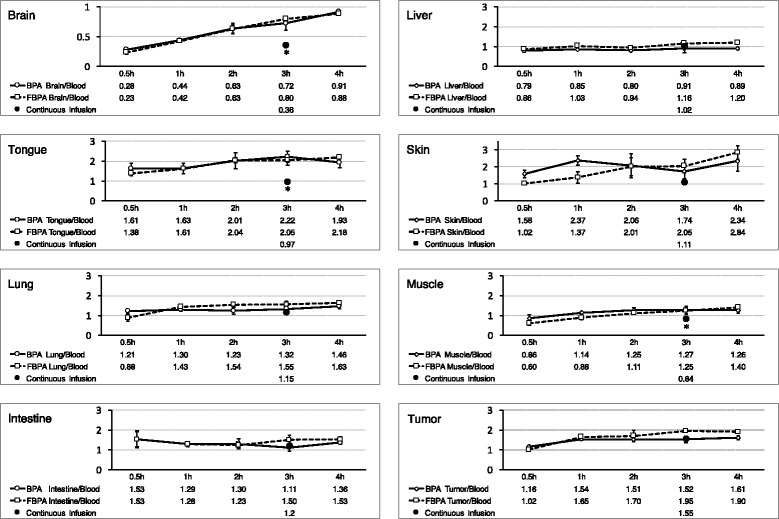
Transition of the tissues to blood ratio of boron concentrations. This figure shows the time transition of each normal tissue to blood ratio and tumour tissue to blood ratio of boron concentrations. The brain to blood ratio increased over time because the boron compounds in the brain were retained 4 h after the injection of L-BPA or [^19^F]-L-FBPA, while the boron in the blood was excreted over time. There was no difference in the transition of the normal tissue to blood ratio and tumour tissue to blood ratio between the L-BPA group and [^19^F]-L-FBPA group. In this figure, the difference between the administration protocols is indicated. The mean boron concentration of the L-BPA group and [^19^F]-L-FBPA group by continuous infusion is denoted as black circles at the time point of 3 h after the start of infusion in this figure. Each continuous infusion value was substituted by the mean value of L-BPA and L-FBPA in continuous infusion protocol. Star signs (*) means significant difference between administration protocols (*p* < 0.05)

**Fig. 4:**
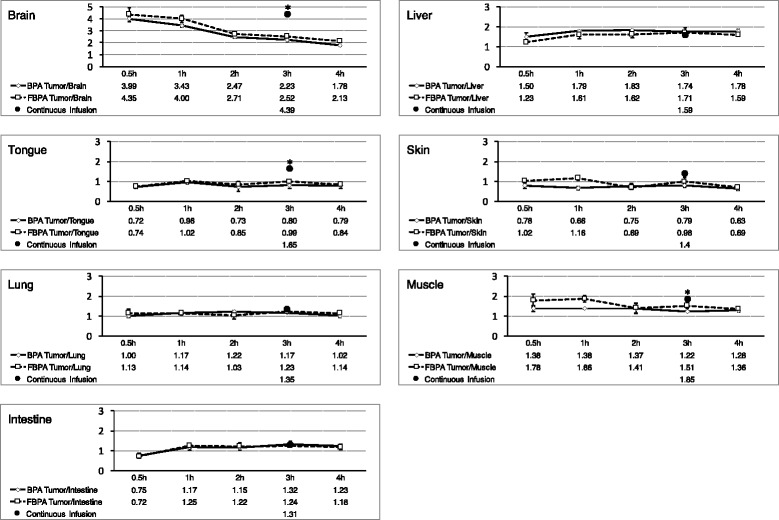
Transition of the tumour tissue to normal tissue ratio of boron concentrations. This figure shows the time transition of each tumour tissue to normal tissue ratio of boron concentrations. There were no differences in the transition of the tumour tissue to normal tissue ratio between the L-BPA group and [^19^F]-L-FBPA group. Each continuous infusion value was substituted by the mean value of L-BPA and L-FBPA in continuous infusion protocol. Star signs (*) means significant difference between administration protocols (*p* < 0.05)

The results of the pharmacokinetics of L-BPA and [^19^F]-L-FBPA by subcutaneous injection are shown in Fig. 2. The maximum values of the blood boron concentration of L-BPA and [^19^F]-L-FBPA were 24.6 and 23.7 μg/g, respectively. The AUC calculated using the linear trapezoidal rule of L-BPA and [^19^F]-L-FBPA were 57.4 μg ∙ hour/g (standard error 2.11) and 49.5 μg ∙ hour/g (standard error 2.32), respectively. Figures 3 and 4 show the transition of the N/B, T/B, and T/N ratios of each normal tissue and tumour tissue. There were no differences in the transition of the N/B, T/B and T/N ratios between the L-BPA group and [^19^F]-L-FBPA group. The 90 % confidence intervals of the maximum concentrations of boron in blood, intestine, liver, lung, tongue, skin, muscle, brain, and tumour were 1.19, 1.47, 1.15, 1.42, 1.30, 1.31, 1.58, 1.10, and 1.24, respectively. The 90 % confidence intervals of AUC in blood, intestine, liver, lung, tongue, skin, muscle, brain, and tumour were 1.33, 1.08, 1.05, 1.08, 1.22, 1.29, 1.13, 1.08, and 1.07, respectively. The 90 % confidence intervals of the maximum concentration of boron in blood were within ranges of 0.80–1.25, while that of AUC in blood were out of the ranges. That of the maximum concentrations and AUC in liver and tumour were within the ranges. However, that of the maximum concentrations in intestine, tongue, and muscle were out of the ranges, while that of AUC in these organs were within the ranges.

### Experiment 2: comparison of the pharmacokinetics of L-BPA and [^19^F]-L-FBPA continuously administered

The boron concentrations of the blood, normal tissue, and tumour tissue after the continuous infusion of L-BPA and [^19^F]-L-FBPA are shown in Table 2. There were no significant differences in the boron concentrations between the L-BPA group and [^19^F]-L-FBPA group. Table 3, Fig. 3 (black circles at the time point of 3 h) show the N/B ratio and T/B ratio of the L-BPA group and [^19^F]-L-FBPA group under the continuous-infusion protocol. No significant difference was observed in the N/B and T/B ratios of the intestine, liver, kidney, tongue, skin, and brain between the L-BPA group and [^19^F]-L-FBPA group. The trend between the L-BPA group and [^19^F]-L-FBPA group was also the same regarding the T/N ratio under the continuous-infusion protocol (Table 4 and Fig. 4).

**Table 2:** The boron concentrations of the blood and tissues under the continuous-infusion protocol

	Blood	Brain	Liver	Tongue	Skin	Lung	Muscle	Intestine	Tumour
L-BPA (μg/g)	13.42 ± 1.26	4.97 ± 0.54	12.11 ± 1.82	13.62 ± 5.15	15.65 ± 2.77	15.82 ± 1.51	12.41 ± 1.19	16.45 ± 2.91	21.11 ± 3.15
L-FBPA (μg/g)	13.19 ± 1.18	4.61 ± 2.28	15.63 ± 3.15	12.14 ± 1.08	13.75 ± 3.08	14.78 ± 1.64	9.69 ± 1.3	15.33 ± 0.88	20.17 ± 6.49
*p* value	0.81	0.81	0.18	0.61	0.45	0.43	0.052	0.51	0.83

**Table 3:** The tissue to blood ratios of boron concentrations under the continuous-infusion protocol

	Intestine	Liver	Kidney	Lung	Tongue	Skin	Muscle	Brain	Tumour
L-BPA (μg/g)	1.22 ± 0.13	0.90 ± 0.09	3.80 ± 1.21	1.18 ± 0.01	1.17 ± 0.167	1.02 ± 0.38	0.93 ± 0.06	0.37 ± 0.05	1.58 ± 0.20
L-FBPA (μg/g)	1.17 ± 0.04	1.18 ± 0.19	4.40 ± 1.04	1.12 ± 0.03	1.03 ± 0.152	0.92 ± 0.01	0.74 ± 0.09	0.35 ± 0.15	1.52 ± 0.42
*p* value	0.46	0.11	0.52	0.048	0.33	0.65	0.044	0.80	0.85

**Table 4:** The tumour tissue to the normal tissue ratios under the continuous-infusion protocol

	Intestine	Liver	Kidney	Lung	Tongue	Skin	Muscle	Brain
L-BPA (μg/g)	1.31 ± 0.26	1.77 ± 0.34	0.46 ± 0.19	1.34 ± 0.16	1.36 ± 0.07	1.64 ± 0.34	1.70 ± 0.14	4.24 ± 0.31
L-FBPA (μg/g)	1.31 ± 0.39	1.34 ± 0.56	0.37 ± 0.15	1.36 ± 0.36	1.47 ± 0.30	1.65 ± 0.44	2.05 ± 0.35	4.60 ± 0.72
*p* value	0.99	0.32	0.51	0.92	0.98	0.58	0.22	0.48

### Experiment 1 and 2: comparison between the single-injection protocol and continuous-infusion protocol

Comparing the single-injection protocol and continuous-infusion protocol, the N/B and T/N ratios of the continuous-infusion group were different from those of the single-injection group (Fig. 3). The N/B ratio of the continuous-infusion protocol was lower than that of the single-injection protocol, while the T/N ratio of the continuous-infusion protocol was higher than that of the single-injection protocol. In contrast, the T/B ratio of the continuous-infusion protocol corresponded with that of the single-injection protocol at 1, 2, and 3 h after L-BPA or [^19^F]-L-FBPA injection (Fig. 3). N/B, T/B, and T/N ratio of liver and tumour tissue had no statistical difference between administration protocols both in L-BPA group and L-FBPA group. On the other hand, N/B and T/N ratio of tongue, muscle, and brain had significant difference between administration protocols both in L-BPA group and L-FBPA group (Figs. 3 and 4).

## Discussion

This study is the first examination comparing the pharmacokinetics of L-BPA and [^19^F]-L-FBPA under the condition of the same dose and protocol (ICP-AES) as that of a mouse model using the cold isotope of [^18^F]-L-FBPA. Previous studies have compared the radioactivity accumulation of [^18^F]-L-FBPA in tumour and normal tissues by PET as well as the boron concentrations of L-BPA in tumour and normal tissues by ICP-AES [16, 20, 21]. Ishiwata and colleagues examined the effect of L-BPA loading on the uptake of [^18^F]-L-FBPA by measuring the radioactivity of ^18^F in B16 melanoma-bearing mice [20]. These authors found that the uptake of [^18^F]-L-FBPA was competitively inhibited by L-BPA loading both in the tumour tissue and muscle. Thus, the uptake mechanism of [^18^F]-L-FBPA is thought to be similar to that of L-BPA. However, the similarities in the the time courses of the N/B and T/N ratios between L-BPA and [^18^F]-L-FBPA remain controversial. Wang et al. examined the difference in the time course after injection of the L-BPA fructose complex and the [^18^F]-L-FBPA fructose complex in a glioma-bearing rat model [21]. These authors found that the T/N ratios of [^18^F]-L-FBPA were higher than those of L-BPA at any time point, although the uptake characteristics of the L-BPA fructose complex and [^18^F]-L-FBPA fructose complex in the tumour tissue were similar, with a maximum observed at 1 h after administration. Hanaoka and colleagues demonstrated a significant correlation between the amount of boron accumulation estimated by PET after injection of the tracer dose of [^18^F]-L-FBPA and that measured by ICP-AES after the injection of 40 mg of the L-BPA fructose complex (*r* = 0.92, *p* < 0.05). However, the experimental conditions of these studies were different regarding the dosage of L-BPA and [^18^F]-L-FBPA (therapeutic dose vs. tracer dose), the method used to measure the boron concentration (ICP-AES vs. PET), and the use of the fructose complex. Additionally, no previous study has compared the single-injection method of L-BPA or [^18^F]-L-FBPA and the continuous-infusion method of these boron compounds, which is the clinical protocol for L-BPA administration used widely in cancer patients. The results of this study showed no apparent difference between L-BPA and [^19^F]-L-FBPA in pharmacokinetics or boron accumulation in tissues. However, certain tendencies were observed between the single-injection protocol and continuous-infusion protocol regarding the N/B, T/B, and T/N ratios between L-BPA and [^19^F]-L-FBPA.

Two aspects in the present study are particularly different from the data of previous studies. First, organs that include air such as the intestine and lung could be evaluated without underestimation by the same protocol in both the L-BPA group and [^19^F]-L-FBPA group in this study. In particular, [^18^F]-L-FBPA accumulation in hollow organs or the lung is significantly underestimated by PET based on the maximum or average counts [16]. In this study, the boron concentrations of the intestine and lung showed no significant difference between the L-BPA group and [^19^F]-L-FBPA group. Second, we compared the pharmacokinetics of two administration protocols: single injection and continuous infusion. L-BPA and [^19^F]-L-FBPA showed the same boron accumulation characteristics in normal and tumour tissues. However, the N/B, T/B and T/N ratios of the continuous-infusion protocol showed different tendencies from those of the single-injection protocol in some organs.

Comparing the single-injection protocol with the continuous-infusion protocol, the T/N ratio by the continuous-infusion protocol was higher than that by the single-injection protocol in many normal tissues such as the brain, tongue, lung, intestine, skin, and muscle (Fig. 3). The N/B ratio in those normal tissues with the continuous-infusion protocol was lower than that by the single-injection protocol measured 1 h after injection. However, the N/B and T/N ratios of the liver and the T/B ratio were almost the same between the continuous-infusion protocol and single-injection protocol (Fig. 3). In this study, we used continuous subcutaneous injection protocol instead of continuous intravenous injection protocol. This is because we concerned the possible effects of anesthesia during continuous intravenous injection to circulation dynamics, blood flow, and tissue distribution of boron compounds. The biological effectiveness of neutron capture therapy depends on the administration dose of L-BPA [17, 22] and the N/B ratio [22, 23]. In this study, we found a difference in the N/B and T/B ratios between the single-injection protocol and continuous-infusion protocol. In applying the N/B or T/N ratios measured by the [^18^F]-L-FBPA PET study to dosimetry in the clinical setting, we should consider the possibility of the difference between administration protocols. Further studies, including pharmacokinetic studies with various animal species, treatment studies using neutron irradiation with different administration protocols, and examinations regarding the difference in the biological effectiveness using the infusion protocol, are needed to presisely estimate the pharmacokinetics in humans and to define both offset values between the values measured by PET and tumour dosimetry in clinical situations and appropriate time points of PET values after the single injection of [^18^F]-L-FBPA.

There are some limitations to this study. First, our measurement methods by ICP-AES could not evaluate the microdistribution of boron atoms in tissue components. GM Morris and colleagues showed the different microdistribution patterns of boron atoms in murine tongue mucosa following the administration of L-BPA or BSH [24]. BNCT is based on the neutron capture reaction resulting in two particles (^4^He and ^7^Li) whose range is limited to the diameter of a single cell (5–9 μm). Therefore, microdistribution impacts both tumour targeting and normal tissue damage. In this study, we did not evaluate microdistribution because we focused on the equivalence of [^19^F]-L-FBPA over L-BPA and because [^18^F]-L-FBPA PET values in clinical settings represent the “macro” distribution similar to the boron concentrations measured by ICP-AES. However, microdistribution should be examined in evaluating treatment results or the equivalence of biological effects rather than the pharmacokinetics of boron compounds [25].

The second limitation is the identicalness of [^18^F]-L-FBPA and [^19^F]-L-FBPA. The change of chemical structure of [^18^F]-L-FBPA may happen by nuclear reaction of ^18^F. This would cause the difference of the pharmacokinetics between [^18^F]-L-FBPA and [^19^F]-L-FBPA. Previous report that studied the metabolism of [^18^F]-L-FBPA in vivo mouse model showed that [^18^F]-L-FBPA was stable to metabolic alteration in plasma and tumour tissue [13]. Thus, we assumed the pharmacokinetics of [^18^F]-L-FBPA and [^19^F]-L-FBPA were identical in this study. The third limitation of our study was the possibility of [^18^F]-L-FBPA metabolization. The cleavage of fluorine from [^18^F]-L-FBPA leads to the underestimation of [^18^F]-L-FBPA accumulation in normal and tumour tissues as measured by PET [20]. In this study, we evaluated the accumulation of [^19^F]-L-FBPA using the same methods as those for the evaluation of L-BPA accumulation. Thus, the metabolization of [^19^F]-L-FBPA did not affect the results of our study. However, we must consider the stability of [^18^F]-L-FBPA and impact of [^18^F]-L-FBPA metabolites in boron accumulation in normal and tumour tissues when the long-term dynamic study of [^18^F]-L-FBPA is scheduled. For instance, a previous study showed the stability of the tracer dose of [^18^F]-L-FBPA for metabolic alterations over 6 h [26]. In addition, if ^18^F is separated from [^18^F]-L-FBPA in other organs such as liver and intestine by some enzymatic metabolism, free ^18^F would be released to blood flow and the deposition of ^18^F to bone should be observed in dynamic PET imaging study in clinical situations as observed in [^18^F]-NaF PET imaging. However, no such image is obtained in dynamic PET study of [^18^F]-L-FBPA in clinical situations. Thus, we believe that an [^18^F]-L-FBPA PET dynamic study with a maximum duration of 6 h can be applicable for the evaluation of L-BPA accumulation in clinical settings.

## Conclusions

We showed the equivalence of the pharmacokinetics of L-BPA and [^19^F]-L-FBPA under the condition of the same fructose complex and dose using the same protocol as that for a subcutaneous tumour mouse model. Next, we evaluated the effects of the administration protocol on the boron distribution in normal and tumour tissues. Some differences were observed between the single-injection protocol and continuous-infusion protocol. In applying the measured values in the F-BPA PET study for dosimetry in BNCT, the possible difference in the N/B and T/N ratios between [^18^F]-L-FBPA single injection and L-BPA continuous infusion should be carefully considered. This study provides useful information for the future clinical application of [^18^F]-L-FBPA PET in clinical dose calculation for BNCT in cancer therapy.

## Additional files

Additional file 1: Figure S1. Synthesis of L-FBPA. (PPTX 101 kb)
Additional file 2: Experimental datasets. (XLSX 62 kb)

## Abbreviations

[^18^F]-L-FBPA: *o*-[^18^F]-fluoro-L-*p*-boronophenylalanine
AUC: Areas under the blood boron concentration curve
BNCT: Boron neutron capture therapy
BSH: Disodium mercaptoundecahydrododecaborate
ICP-AES: Inductive coupled plasma atomic emission spectrometry
L-BPA: L-*p*-boronophenylalanine
N/B ratio: The normal tissue to blood ratio of boron concentrations
PET: Positron emission tomography
T/B ratio: The tumour to blood ratio of boron concentrations
T/N ratio: The tumour to the normal tissue ratio of boron concentrations
