# Supplementary material for: Comparison of the pharmacokinetics between L-BPA and L-FBPA using the same administration dose and protocol: a validation study for the theranostic approach using [18F]-L-FBPA positron emission tomography in boron neutron capture therapy
Source: BMC Cancer. 2016 Nov 8;16:859. doi: 10.1186/s12885-016-2913-x (PMC5100278; doi:10.1186/s12885-016-2913-x)
Supplement: Additional file 1: Figure S1. — Synthesis of L-FBPA. (PPTX 101 kb) [file 12885_2016_2913_MOESM1_ESM.pptx]

## Slide 1
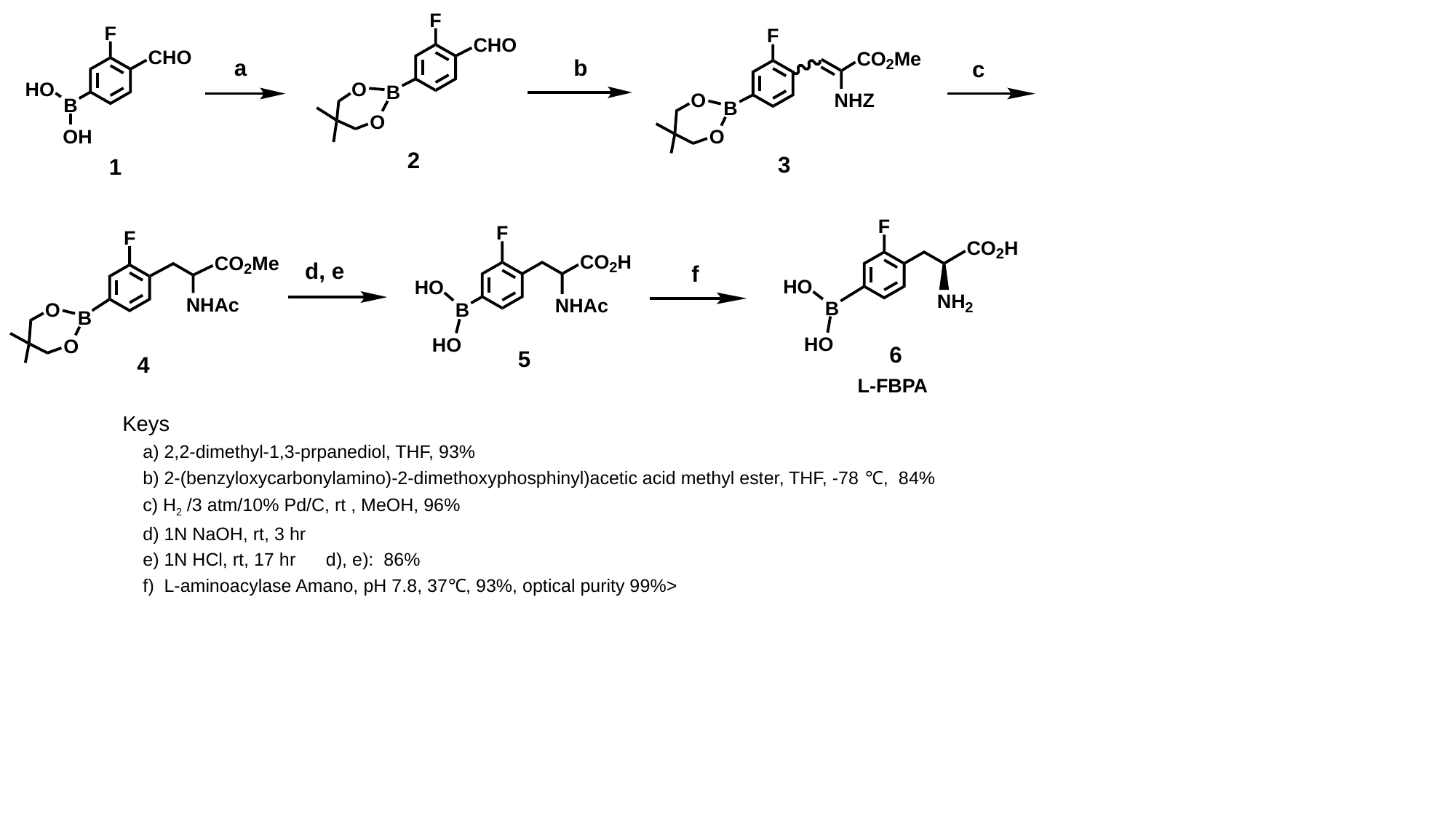

Keys
 a) 2,2-dimethyl-1,3-prpanediol, THF, 93%
 b) 2-(benzyloxycarbonylamino)-2-dimethoxyphosphinyl)acetic acid methyl ester, THF, -78 ℃, 84%
 c) H2 /3 atm/10% Pd/C, rt , MeOH, 96%
 d) 1N NaOH, rt, 3 hr
 e) 1N HCl, rt, 17 hr d), e): 86%
 f) L-aminoacylase Amano, pH 7.8, 37℃, 93%, optical purity 99%>
